# Scalp, Oral, and Nail Pemphigus Vulgaris: Clinical Characteristics and a Review of the Literature

**DOI:** 10.7759/cureus.38334

**Published:** 2023-04-30

**Authors:** Emily Eachus, Lauren E DeLamielleure, Samrah Mitha, Taha F Rasul, Arfa Faiz

**Affiliations:** 1 Medical Education, University of Miami Miller School of Medicine, Miami, USA; 2 Biomedical Sciences, Florida Atlantic University Charles E. Schmidt College of Medicine, Boca Raton, USA; 3 Geriatrics, Nova Southeastern University, Davie, USA; 4 Allergy and Immunology, Sutter Medical Center, Sacramento, USA

**Keywords:** immuno-histochemical, allergic dermatoses, nail diseases, scalp condition, pemphigus vulgaris

## Abstract

Pemphigus vulgaris (PV) is a rare disease that affects the skin and mucous membranes, causing blistering and erosions. Identifying and effectively managing atypical presentations of pemphigus vulgaris can be challenging due to its rarity. We describe a 32-year-old male patient with a medical history including prediabetes, moderate asthma, hyperlipidemia, coccidioidomycosis, and respiratory infections. He was evaluated via telehealth in the allergy and immunology clinic for uncontrolled asthma. Initially, he complained of a whitish film in the mouth while on treatment with fluticasone and salmeterol. He also noted new vesicular lesions on his scalp and body. When evaluated later in the clinic, he was found to have oral and periungual erosions as well as paronychia. After promptly referring to dermatology, histopathological examination and direct immunofluorescence testing were performed on the patient's lesions, revealing changes consistent with PV. Treatment with prednisone and rituximab resulted in the complete resolution of the patient's bullae and nail deformities over several months. This case highlights the importance of a thorough evaluation of complex medical histories and diagnostic testing in managing asthma and allergy symptoms. It also emphasizes the need for a multidisciplinary approach involving specialists such as immunologists, dermatologists, and infectious disease experts in the diagnosis and management of complex cases.

## Introduction

Pemphigus vulgaris (PV) is a rare autoimmune disease that can cause cutaneous and mucosal blisters. It has a prevalence of between 0.1 and 0.5 per 100,000, with a higher percentage of cases in people of Ashkenazi Jewish descent [1]. In 80% of patients, the initial blistering occurs in the oral mucosa, and in 75% of patients, cutaneous manifestations follow after the first oral mucosal blister. The autoimmune component consists of autoantibodies that target keratinocyte proteins, causing acantholysis due to the loss of keratinocyte-to-keratinocyte interactions [1]. After the initial presentation, both the oral and mucosal blisters frequently turn into erosions. Cutaneous lesions are characterized by flaccid blisters that can progress into erosions and eventually form crusts [2]. The oral lesions in PV can mimic many diseases, such as mucous membrane pemphigoid, systemic lupus erythematosus, and lichen planus [2].

The diagnosis of pemphigus vulgaris is done via biopsy of a normal-appearing perilesional area of the skin or mucosa. The sample is analyzed using direct immunofluorescence (DIF). Tzanck smears and enzyme-linked immunosorbent assay (ELISA) can also be used to confirm the diagnosis. The first-line treatment for pemphigus vulgaris is systemic corticosteroids. If corticosteroids are not decreasing lesion severity, azathioprine, or mycophenolate mofetil (MMF) can be added to the regimen [3].

Atypical presentations of PV are rare but can occur. These include pemphigus vegetans, pemphigus herpetiformis, and paraneoplastic pemphigus [4]. Pemphigus vegetans presents as hypertrophic vegetating lesions in intertriginous areas, while pemphigus herpetiformis is characterized by a vesicular eruption that resembles dermatitis herpetiformis. Paraneoplastic pemphigus is a rare variant associated with an underlying malignancy and can present with a polymorphic clinical picture that involves both mucosal and cutaneous areas. However, atypical PV can also appear based solely on location, such as in the case of our patient. This report describes an atypical anatomic presentation of PV with simultaneous scalp, nail, skin, and oral involvement.

## Case presentation

**Figure 1:**
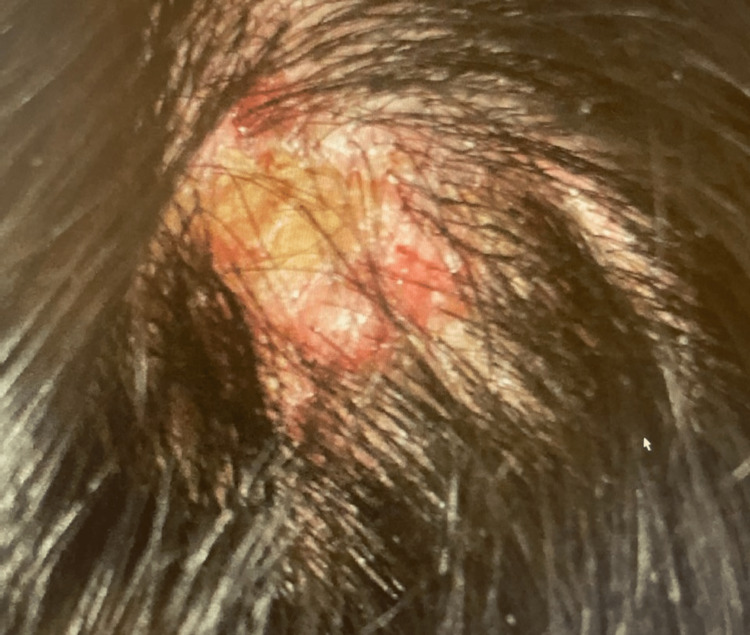
A blistering lesion with surrounding erosions on the patient's scalp.

A 32-year-old male with a history of prediabetes, mild asthma, hyperlipidemia, and smoking presented for a consultation at the allergy and immunology clinic to discuss his asthma and allergy symptoms. He reported that his asthma had started two years prior and had been of mild severity, While the symptoms were initially bothersome, they were usually manageable with short-acting beta agonists. His most recent serological test came back positive for "desert fever" (coccidioidomycosis). During the examination, fresh-appearing lesions on the scalp were identified (Figure 1).

The patient had been using fluticasone and salmeterol to treat his asthma; however, it was ineffective and contributed to the development of oropharyngeal candidiasis which was noted at the initial appointment. In order to avoid the adverse effects of fluticasone, he switched to ciclesonide at the first appointment. Ciclesonide proved better at managing his symptoms of cough and shortness of breath four weeks later. He also had a history of smoking and recently tested positive for coronavirus disease 2019 (COVID-19), about three weeks prior.

During the consultation, the patient's laboratory, imaging, and diagnostic evaluations revealed positive immunoglobulin (Ig)M titers for coccidioidomycosis. The patient's initial complaint of "desert fever" and geographic location in the Southwestern United States prompted the IgM titers. The patient's medical history suggested a complex case of asthma exacerbated by recent COVID-19 infection and complications related to medication use, such as candida caused by fluticasone and salmeterol. The positive IgM titers for coccidioidomycosis indicated the presence of an infectious agent, requiring further evaluation and treatment. He did not have other symptoms consistent with coccidioidomycosis at the time. An expedited referral was made to an infectious disease specialist due to concerns of the lesions having infectious etiology. The infectious disease specialist expressed doubt regarding the infectious etiology of the cutaneous lesions. Nevertheless, a six-month course of fluconazole was initiated. The patient's condition deteriorated further while on this treatment, necessitating a re-evaluation.

**Figure 2:**
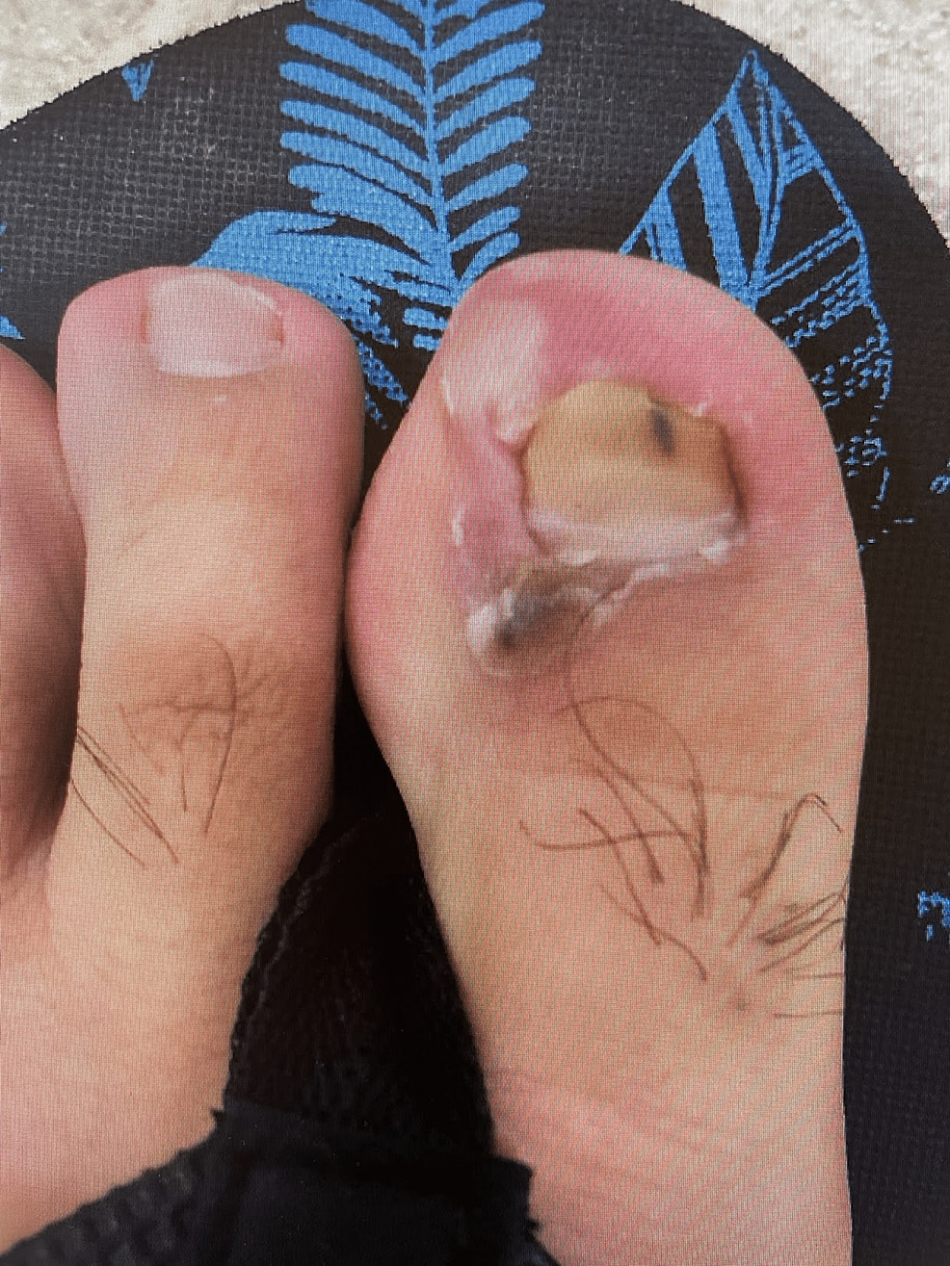
Left toe with an erosion adjacent to the nail fold and paronychia.

At the four-week follow-up visit, the patient reported worsening of scalp and oral lesions and episodes of "white stuff scraping off" his tongue. Fluconazole and an oral mouthwash (a combination of diphenhydramine, hydrocortisone, and nystatin) were prescribed but showed little improvement. The lesions spread to the patient's abdomen and extremities, necessitating a dermatology visit. Physical examination revealed flaccid bullae and erosions on the extremities, trunk, and face. Additionally, multiple periungual bullae with surrounding erythema were noted on the fingers and toes (Figure 2).

**Figure 3:**
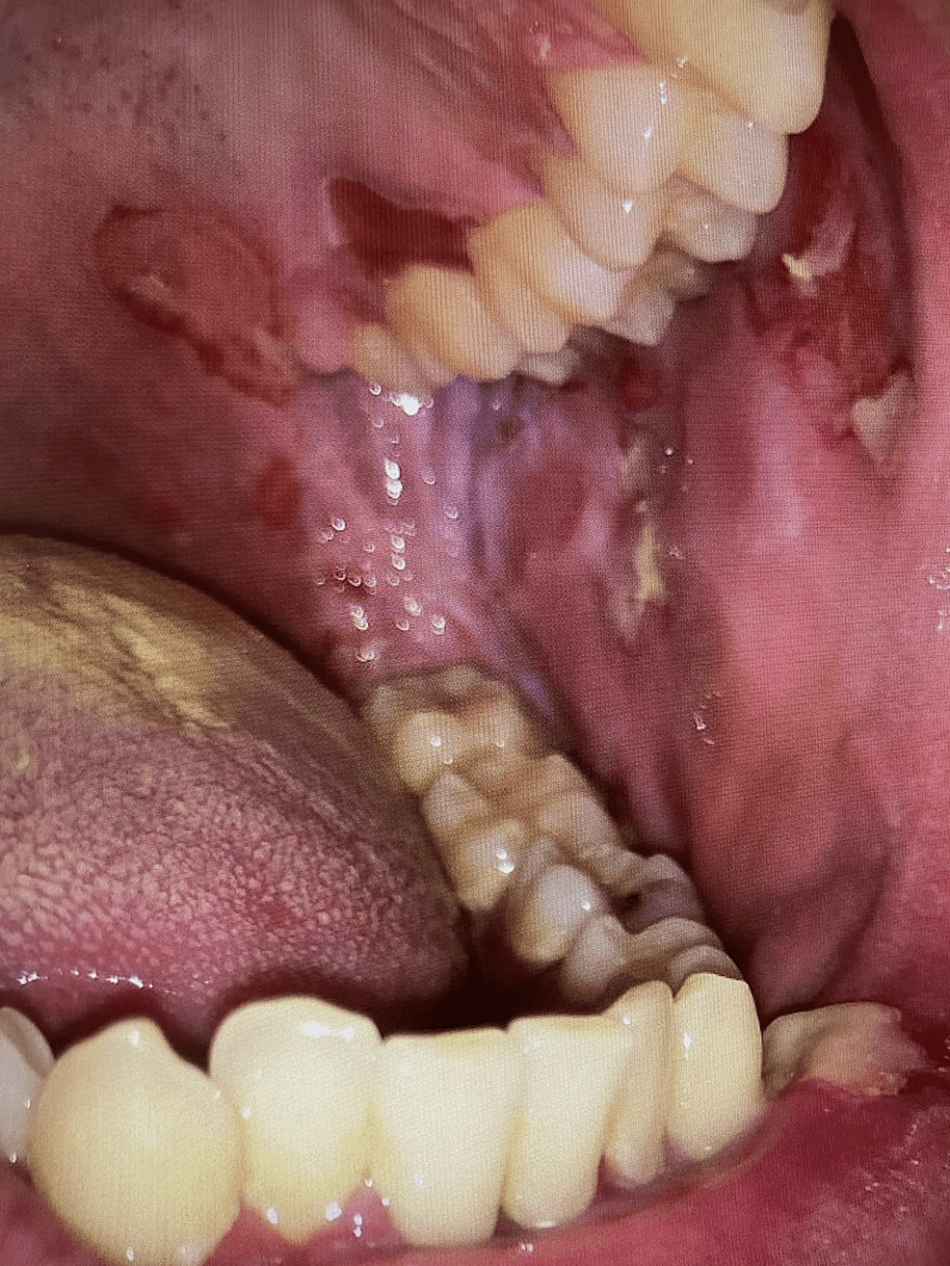
Oral erosions and lesions. Note the candidiasis present on the tongue surface.

The oral mucosa was found to have painful and fragile erosions affecting the lips, tongue, buccal mucosa, and gingiva. These erosions were shallow and partially covered by a white or yellowish pseudomembrane (Figure 3). When the pseudomembrane was removed, an underlying raw and bleeding surface was exposed.

Perilesional biopsies were performed in the office to examine the lesions. The histopathology results demonstrated suprabasal acantholysis, and direct immunofluorescence revealed a "chicken wire" pattern of IgG deposition along the intercellular spaces of the epidermis, which reflected the autoantibodies targeting desmoglein 1 and 3. The levels of desmoglein 1 and 3 antibodies were measured, with values of 142 U/mL and 153 U/mL, respectively. A positive desmoglein test is indicated by a value greater than 20 U/mL, and both results were significantly higher than this threshold.

BP180 autoantibody and BP230 IgG were both negative, with levels of less than 5 U/mL and less than 9 U/mL, respectively. The patient was given a preliminary diagnosis of pemphigus vulgaris and prescribed 60 mg of prednisone daily. Although the treatment improved some of his oral lesions, it did not show significant improvement in his skin, scalp, and nail manifestations.

Following a second biopsy of the right buccal erythroplakia, direct immunofluorescence revealed intracellular IgG staining indicative of a condition similar to pemphigus. The final pathology workup confirmed the presence of pemphigus vulgaris-conforming acantholytic dermatosis, although the final unifying diagnosis was PV. Due to a lack of response on the scalp and nails, the patient was scheduled for a rituximab infusion and prescribed prednisone tablets of 100 mg once daily, along with doxycycline as a prophylactic.

Over several months, the patient's condition improved, with complete resolution of the bullae and nail deformities. The patient continued to receive regular follow-up appointments at the dermatology, allergy and immunology, and infectious diseases departments.

## Discussion

This case highlights an atypical presentation of pemphigus vulgaris as an oozing scalp lesion with simultaneous nail involvement, while also showing a case where oral lesions of PV were initially mistaken as uncomplicated thrush. A literature review revealed other atypical sites for PV presentations: the vulva and cervix, the esophagus, nails, the umbilical region, and the nose (Table 1).

**Table 1:** Eight case reports that highlight unusual presentations of pemphigus vulgaris. PV, Pemphigus Vulgaris; EDS, esophagitis dissecans superficialis

Author and Year of Publication	Design	Findings	Treatment
Smith et al., 2021 [4]	Case Report	Two cases of pemphigus vulgaris in women presented with associated postmenopausal bleeding. The site of bleeding was established to be related to cervical involvement in one patient and assumed to be of cervical origin in the latter.	The first patient was initially treated with prednisone, and later opted for a hysterectomy. After another flare of PV, she was given rituximab therapy. The second patient was only treated with corticosteroids.
Maldonado-Paredes et al., 2022 [5]	Case Report	A 37-year-old female presented to a gastrointestinal clinic for weight loss secondary to dysphagia, odynophagia and retrosternal pain. An endoscopy was performed which showed esophagitis dissecans superficialis (EDS) and erythematous gastropathy of the antrum. These findings indicated pemphigus vulgaris with exclusive involvement of the esophagus.	This patient’s esophageal presentation of PV was managed with deflazacort, azathioprine, omeprazole, and sucralfate.
Kaneoka et al., 2022 [6]	Case Report	A 73-year-old male presented with an erosion on his nose that he contributed to wearing a face mask. A skin biopsy showed acantholysis in the epidermal granular layer. The friction from wearing the mask was thought to be a factor in the exacerbation of PV in this patient.	This patient responded positively to oral prednisolone and azathioprine.
Gupta et al., 2003 [7]	Case Report	A cervical pap smear from a 52-year-old female revealed cytomorphologic features that were consistent with acantholytic cells of pemphigus.	Unavailable
Thakur et al., 2020 [8]	Case Report	Oral manifestations of PV in the uncommon locations such as the attached, marginal, and interdental gingiva with no involvement of oral mucosa, tongue, palate, or buccal mucosa in a 32-year-old patient. Acantholytic cells were present in the cytological smear; however, no biopsy was performed to confirm diagnosis of PV.	.75-1 mg/kg/day of prednisolone and topical steroids such as clobetasol propionate were recommended. Periodontal care involves the use of .2% chlorhexidine mouth rinses and routine electronic toothbrush use at home.
Kolivras et al., 2003 [9]	Case Report	A 69-year-old female with an 11-year history of PV presented with an erosive right hallux nail bed lesion, accompanied by nail plate destruction, subungual hyperkeratosis, and paronychia. Mucocutaneous lesions were also present.	Initial phase involved two rounds of plasmapheresis followed by a prednisolone 100 mg/day taper as well as azathioprine 50 mg/day.
Patsatsi et al., 2009 [10]	Case Report	A 60-year-old woman had painful erosions in her mouth, perineum, and anal area, thick crusty plaques on her scalp, and discolored fingernails. Her toenails were inflamed, bleeding, and had hemorrhages under or within them. Doctors diagnosed her with PV based on lab tests and observation of acantholytic cells in a subungual lesion.	Oral corticosteroids at 80 mg/day were tapered down to 5 mg/day. Topical mupirocin ointment was applied for 10 days to the nail folds and visible lesions.
Moussaoui et al., 2021 [11]	Case Report	A Female patient was diagnosed with PV that begun with simultaneous oral and umbilical locations which coexisted for a period of four months before the appearance of other skin lesions.	Daily intravenous administration of prednisolone (120 mg/day) and amoxicillin-clavulanic acid (1000 mg thrice a day) was started. The nursing team was instructed to clean the oral cavity and the umbilicus using chlorhexidine (0.12%) mouthwash and ointment. Her condition improved gradually during hospitalization. There was a complete remission of the lesions in the mouth and umbilicus. The patient was advised to continue oral prednisone intake at a dose of 20 mg/day.

Pemphigus vulgaris (PV) is a rare autoimmune disease that affects the skin and mucous membranes and is characterized by the development of blisters and erosions. The classic presentation of PV involves the oral cavity, with the appearance of painful blisters and erosions on the mucosa, followed by the development of skin lesions. However, PV can also have atypical presentations that may lead to a delayed or missed diagnosis [4-11]. These can be structural (pemphigus vegetans) or anatomic (scalp and nail pemphigus).

One atypical presentation of PV is the absence of oral involvement [12]. In some cases, patients may present with only cutaneous lesions, which may be mistaken for other dermatological conditions, such as bullous pemphigoid, dermatitis herpetiformis, or erythema multiforme. These patients may also have less rapid disease activity, making the diagnosis more difficult. Another atypical presentation of PV is the involvement of the scalp. This may present as crusted, oozing, and pruritic lesions, which may mimic other scalp conditions, such as seborrheic dermatitis, psoriasis, or tinea capitis [13]. Diagnosis can be further complicated by the fact that scalp involvement is less common in PV than in other autoimmune blistering diseases.

When a patient is suspected to have PV, the non-dermatology specialist can perform a thorough medical history and physical examination, as well as order diagnostic tests to screen for PV [14]. These diagnostic tests include skin biopsies for histopathological examination and direct immunofluorescence (DIF) to detect autoantibodies against desmoglein 1 and 3. The pathophysiology of PV is often characterized by the destruction of desmoglein (Dsg1) and desmoglein 3 (Dsg3) by IgG autoantibodies and histopathological findings revealing intraepithelial split with detached keratinocytes (acantholysis). Dsg1 and Dsg3 are components of desmosomes, which function to hold together the mucous membrane and skin epithelial cells. Dsg3 is primarily expressed in the mucosal epithelium, whereas desmoglein 1 is found in the epidermis and superficial layers of the skin. The targeting of these molecules results in the loss of cell adhesion and consequential intraepithelial cleavage (suprabasilar split) [15]. Blood tests to detect circulating autoantibodies, such as anti-desmoglein antibodies, can also aid in the diagnosis of PV and often have high sensitivity.

In some cases, PV may present as a drug-induced rash when a patient develops PV after exposure to a medication that triggers an autoimmune response. Medications associated with drug-induced PV include penicillamine, captopril, and rifampin [16]. It can be challenging to identify PV as the rash may resemble a drug reaction, and the onset of PV may occur weeks or months after the initiation of the offending medication. Finally, PV may also present as a paraneoplastic syndrome, meaning that it occurs in association with an underlying malignancy. The most commonly associated malignancies are hematologic cancers, such as non-Hodgkin lymphoma and chronic lymphocytic leukemia [17]. However, PV diagnosis can be delayed or missed in these cases, as treatment efforts are primarily directed towards the underlying malignancy. Diagnosis of PV can be elusive due to its varied presentation. A thorough medical history, including medication use and malignancy screening, is essential in the evaluation of patients with suspected PV.

If PV is confirmed, the physician can develop a treatment plan tailored to the patient's individual needs. The usual course of treatment for PV generally entails the use of immunosuppressive drugs, such as corticosteroids, rituximab, or azathioprine; however, these medications can cause significant side effects and necessitate frequent monitoring [16]. The successful management of the extensive and debilitating skin and mucosal lesions commonly seen in pemphigus vulgaris (PV) relies heavily on the specialized knowledge and expertise of dermatologists. The dermatologist may perform skin examinations, repair wounds, and administer topical or systemic medications to manage the cutaneous symptoms associated with PV. By working in tandem, allergy/immunology specialists and dermatologists can effectively monitor disease activity and response to treatment, tailor medications as necessary, and promptly address any complications or side effects that arise. This comprehensive, multidisciplinary approach not only ensures optimal care for patients with PV but also enhances their overall quality of life by providing coordinated, streamlined care that is tailored to their unique needs and circumstances.

## Conclusions

Pemphigus vulgaris is a rare, autoimmune condition that can present with lesions on atypical areas such as the scalp and nails. Our patient's presentation was unusual in terms of initial presenting symptoms such as cough and infection as well as simultaneous oral, nail, scalp, and skin involvement. A definitive diagnosis was made based on histopathological and immunofluorescence findings. The patient's initial treatment with prednisone improved his oral lesions. Rituximab infusions were added to manage his skin, scalp, and nail lesions.

The heterogenous presentation of symptoms and the varied initial locations associated with pemphigus vulgaris can pose diagnostic challenges. Thorough assessments of clinical, histological, immunological, and serological findings should be utilized for patients with skin conditions accompanied with or without simultaneous oral lesions to avoid delayed or missed diagnoses. Comprehensive treatment and improved outcomes for those suffering from this rare autoimmune condition can be achieved through multispecialty collaboration.

## References

[1] Firooz A, Mazhar A, Ahmed AR (1994). Prevalence of autoimmune diseases in the family members of patients with pemphigus vulgaris. J Am Acad Dermatol.

[2] Patel F, Wilken R, Patel FB (2017). Pathophysiology of autoimmune bullous diseases: nature versus nurture. Indian J Dermatol.

[3] Kridin K (2018). Emerging treatment options for the management of pemphigus vulgaris. Ther Clin Risk Manag.

[4] Smith SM, Moscarelli R, Panse G, Parkash V (2021). Cervical pemphigus vulgaris presenting as postmenopausal bleeding. Int J Gynecol Pathol.

[5] Maldonado-Paredes SE, Juárez-Cedillo T, Godínez-Escobar KJ, Contreras-Rodríguez Y, Gallegos-De Luna CF, Alanis-Ocádiz A (2022). Pemphigus vulgaris with exclusive affectation in the esophagus: a case report. Rev Med Inst Mex Seguro Soc.

[6] Kaneoka A, Sawada Y (2022). A case of pemphigus vulgaris showing a local nose erosion as the first clinical manifestation (Article in Japanese). J UOEH.

[7] Gupta S, Sodhani P, Jain S (2003). Acantholytic cells exfoliated from pemphigus vulgaris of the uterine cervix. A case report. Acta Cytol.

[8] Thakur N, Rayast D, Negi M, Bansal S (2020). Pemphigus vulgaris: a rare case of gingival involvement. Contemp Clin Dent.

[9] Kolivras A, Gheeraert P, André J (2003). Nail destruction in pemphigus vulgaris. Dermatology.

[10] Patsatsi A, Sotiriou E, Devliotou-Panagiotidou D, Sotiriadis D (2009). Pemphigus vulgaris affecting 19 nails. Clin Exp Dermatol.

[11] Moussaoui E, Oueslati Y, Oualha L, Denguezli M, Sriha B, Douki N (2021). Simultaneous oral and umbilical locations as a first sign of pemphigus vulgaris. Case Rep Dent.

[12] Gualtieri B, Marzano V, Grando SA (2021). Atypical pemphigus: autoimmunity against desmocollins and other non-desmoglein autoantigens. Ital J Dermatol Venerol.

[13] Mergler R, Kerstan A, Schmidt E, Goebeler M, Benoit S (2017). Atypical clinical and serological manifestation of pemphigus vegetans: a case report and review of the literature. Case Rep Dermatol.

[14] Vassileva S, Drenovska K, Manuelyan K (2014). Autoimmune blistering dermatoses as systemic diseases. Clin Dermatol.

[15] Ghaedi F, Etesami I, Aryanian Z (2021). Drug-induced pemphigus: a systematic review of 170 patients. Int Immunopharmacol.

[16] Porro AM, Seque CA, Ferreira MC, Enokihara MM (2019). Pemphigus vulgaris. An Bras Dermatol.

[17] Pollmann R, Schmidt T, Eming R, Hertl M (2018). Pemphigus: a comprehensive review on pathogenesis, clinical presentation and novel therapeutic approaches. Clin Rev Allergy Immunol.

